# 1894. Sensitivity of Symptom-Based Screening for COVID-19 in Active Duty Service Members

**DOI:** 10.1093/ofid/ofac492.1521

**Published:** 2022-12-15

**Authors:** Zachary Matthews, Daniel Cybulski, Dianne Frankel, John Kieffer, Theresa Casey, Angela Osuna, Joseph E Marcus

**Affiliations:** Uniformed Services University of the Health Sciences, Bethesda, Maryland; Department of Medicine at Brooke Army Medical Center, San Antonio, Texas; Trainee Health Surveillance, San Antonio, Texas; 559th MDG/THLS Trainee Health, JBSA Lackland, Texas; JBSA Lackland Texas, JBSA Lackland, Texas; JBSA Lackland, JBSA Lackland, Texas; Infectious Disease - Brooke Army Medical Center, San Antonio, TX, San Antonio, Texas

## Abstract

**Background:**

Symptomatic COVID-19 screening has been a cornerstone of case identification during the pandemic. Despite the myriad of COVID-19 symptoms, screens have focused on fever, cough, and dyspnea. It is unknown how well these symptoms identify cases in a healthy military population. This study aims to evaluate the utility of symptom-based screening in identifying COVID-19 through different COVID-19 waves.

**Methods:**

A convenience sample of 600 active-duty service members who arrived at JBSA in 2021 and 2022 was included in this study. We compared 200 symptomatic service members who tested positive for COVID-19 in each of FEB-APR 2021 (prior to the emergence of the Delta variant), JUN-AUG 2021 (Delta variant was predominant), and JAN 2022 (Omicron variant was predominant). Collected data included test date, reported symptoms, and vaccination status. Comparisons were conducted via Chi-Square or Fisher’s Exact test.

**Results:**

Of the 600 symptomatic active-duty service members who tested positive for COVID-19, the most common symptoms were sore throat (n=385, 64%), headache (n=334, 56%), and cough (n=314, 52%). While sore throat was the most prominent symptom during Delta (n=140, 70%) and Omicron (n=153, 77%), headache was the most common prior to Delta (n=93, 47%). There were significant differences in symptoms by vaccination status (Table 2). Overall, screening for fever, cough, and dyspnea had a 65.1% sensitivity in this cohort (Table 3) with its lowest sensitivity in the pre-Delta cohort (53.5%) and highest sensitivity in the fully vaccinated Omicron cohort (78.3%).

**Figure:**
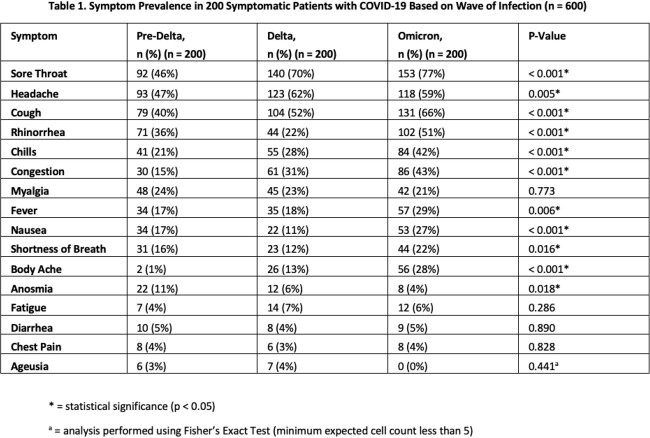

**Figure:**
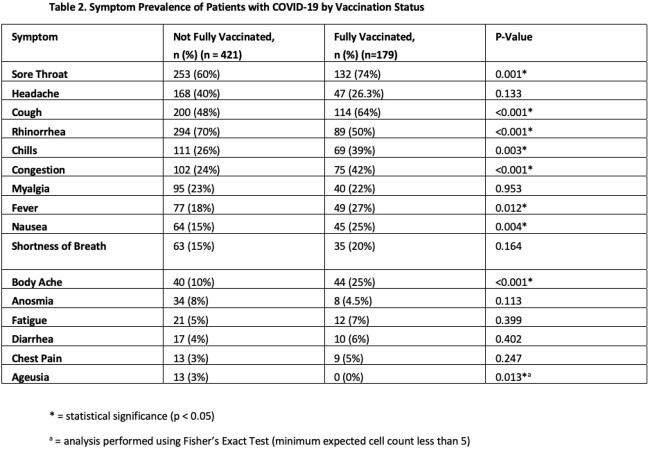

**Figure:**
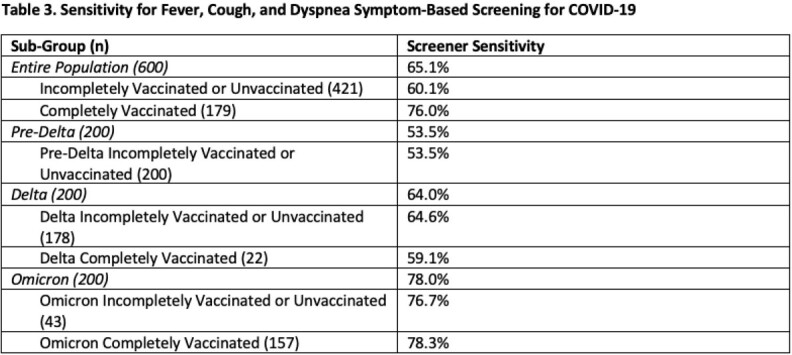

**Conclusion:**

In this descriptive cross-sectional study evaluating symptomatic military members with COVID-19, symptom prevalence varied based on the predominant COVID-19 variant as well as patients’ vaccination status. As screening strategies evolve with the pandemic, changing symptom prevalence should be considered.

**Disclosures:**

**All Authors**: No reported disclosures.

